# Nonerythropoietic Erythropoietin-Derived Peptide Suppresses Adipogenesis, Inflammation, Obesity and Insulin Resistance

**DOI:** 10.1038/srep15134

**Published:** 2015-10-13

**Authors:** Yuqi Liu, Bangwei Luo, Rongchen Shi, Jinsong Wang, Zongwei Liu, Wei Liu, Shufeng Wang, Zhiren Zhang

**Affiliations:** 1 Institute of Immunology, Third Military Medical University, 30 Gaotanyan Main Street, Chongqing 400038, People’s Republic of China

## Abstract

Erythropoietin (EPO) has been identified as being crucial for obesity modulation; however, its erythropoietic activity may limit its clinical application. EPO-derived Helix B-surface peptide (pHBSP) is nonerythrogenic but has been reported to retain other functions of EPO. The current study aimed to evaluate the effects and potential mechanisms of pHBSP in obesity modulation. We found that pHBSP suppressed adipogenesis, adipokine expression and peroxisome proliferator-activated receptor γ (PPARγ) levels during 3T3-L1 preadipocyte maturation through the EPO receptor (EPOR). In addition, also through EPOR, pHBSP attenuated macrophage inflammatory activation and promoted PPARγ expression. Furthermore, PPARγ deficiency partly ablated the anti-inflammatory activity of pHBSP in macrophages. Correspondingly, pHBSP administration to high-fat diet (HFD)-fed mice significantly improved obesity, insulin resistance (IR) and adipose tissue inflammation without stimulating hematopoiesis. Therefore, pHBSP can significantly protect against obesity and IR partly by inhibiting adipogenesis and inflammation. These findings have therapeutic implications for metabolic disorders, such as obesity and diabetes.

Obesity is a global and persistent growing health epidemic, whose incidence has almost doubled during the last 30 years^1^,^2^. Increased energy intake or decreased energy expenditure will lead to a massive increase in adipose tissue^3^, increasing the risk of type 2 diabetes (T2D) and other chronic diseases^4^. The prevention of obesity involves both dietary factors and physical activities; however, the incidence of obesity is still rising, suggesting that more attention should be focused on discovering new therapies^5^.

Obesity involves the formation of new adipocytes from precursor cells (adipocyte hyperplasia) and an increase in adipocyte size (adipocyte hypertrophy). In addition, adipocytes also secrete a variety of fatty acids and adipokines when they increase in size, which are closely associated with obesity-associated chronic diseases, such as T2D and cardiovascular disease^6^. Adipocyte hypertrophy is the main cause of adult-onset obesity, whereas adipocyte hyperplasia can be observed in children and morbidly obese adults^7^. Therefore, the modulation of adipocyte hypertrophy and hyperplasia can be important for obesity intervention^8^.

Obesity is usually related to a chronic, low-grade inflammatory response, which is initiated by excess nutrients in metabolic cells and eventually exacerbated by further activation of specialized immune cells^9^. Growing evidence has established the causative links between obesity-induced inflammation and obesity-related insulin resistance (IR)^10^. For example, the proinflammatory cytokine TNF-α has been proven to mediate obesity-induced IR in rodents, and the chemokine monocyte chemotactic protein-1 (MCP-1) has been demonstrated to impair adipocyte insulin sensitivity^11^,^12^. Many cells are involved in obesity-associated inflammation, among which macrophages play an essential role. Adipose tissue macrophages (ATMs) comprise almost 40% of the immune cells in obese adipose tissue, playing key roles in regulating systemic IR, glucose tolerance and the development of metabolic dysfunction^13^. Obesity induces the activation of proinflammatory signaling in ATMs, resulting in upregulation of pro-inflammatory cytokines (i.e., TNF-α, IL-6 and inducible nitric oxide synthase (iNOS)), which act locally, contributing to IR^14^. Furthermore, the inhibition of inflammatory pathways in obesity has beneficial effects on insulin sensitivity in mouse models and human trials^15^,^16^,^17^.

Erythropoietin (EPO) is a pleiotropic hormone that regulates the production of red blood cells by binding to homodimer EPO receptor (EPOR_2_) and has been widely employed to treat anemia^18^. Moreover, a growing number of studies have reported EPOR expression in different cells, such as adipocytes, macrophages, neurons, endothelial cells and cardiac cells^19^,^20^,^21^,^22^. In adipocytes, EPO has been found to decrease preadipocyte differentiation, and mice with adipocyte-specific deletion of EPOR exhibited obesity and decreased glucose tolerance and insulin sensitivity^23^. In macrophages, EPO attenuates LPS-induced expression of IL-6 and TNF-α^9^. More importantly, exogenous EPO has been deemed to improve obesity and IR, indicating that EPO is a potent regulator of obesity^24^. However, EPO can induce erythropoiesis, and its long-term application may cause side effects, such as increasing hematocrit, raising blood pressure and increasing the risk of thrombosis, which limit its long-term clinical application^25^. Fortunately, an increasing number of studies have revealed that the tissue-protective function of EPO is induced via the activation of the EPOR-CD131 complex, the so-called tissue-protective receptor (TPR), whose affinity for EPO is 100 times lower than that of the homodimer EPOR_2_ 26. Additionally, EPO analogues that are reported to maintain the tissue-protective properties of EPO while lacking erythropoietic potential have been developed.

EPO is determined to interact with (EPOR)_2_ through its 3D structure (helix A, C and D). However, helix B is considered to exert the selective tissue-protective features of EPO. pHBSP is an EPO Helix B-derived short peptide of 11 amino acids in length, which was reported to have no erythropoietic activity while retaining the tissue-protective properties of EPO and selectively binding to the EPOR-CD131 complex^27^,^28^. To better understand this EPO analogue, we constructed a stick model of pHBSP (Supplementary Figure 1). Although pHBSP has a short plasma half-life of several minutes, its protective effects in animals and humans can last over hours to days, indicating a rapid passage of pHBSP into affected compartments and a rapid activation of TPR, leading to the sustained activation of related signal pathways, such as the PI3K/Akt axis^29^. Recently, pHBSP has been reported to promote recovery from various diseases, such as burn injury, stroke, wound healing, renal ischemia/reperfusion, autoimmune diseases and status epileptics, and it has also been reported to improve diet-induced IR. In addition, accumulated results from preclinical toxicological studies have raised no safety concerns regarding the application of pHBSP^30^,^31^,^32^,^33^,^34^. However, the long-term effects and potential mechanisms of pHBSP on obesity, IR and inflammation are not fully understood. Therefore, in the current study, we investigated the effects and potential mechanisms of pHBSP on obesity regulation.

## Results

### pHBSP inhibits adipogenesis in 3T3-L1 preadipocytes through TPR

**Figure 1:**
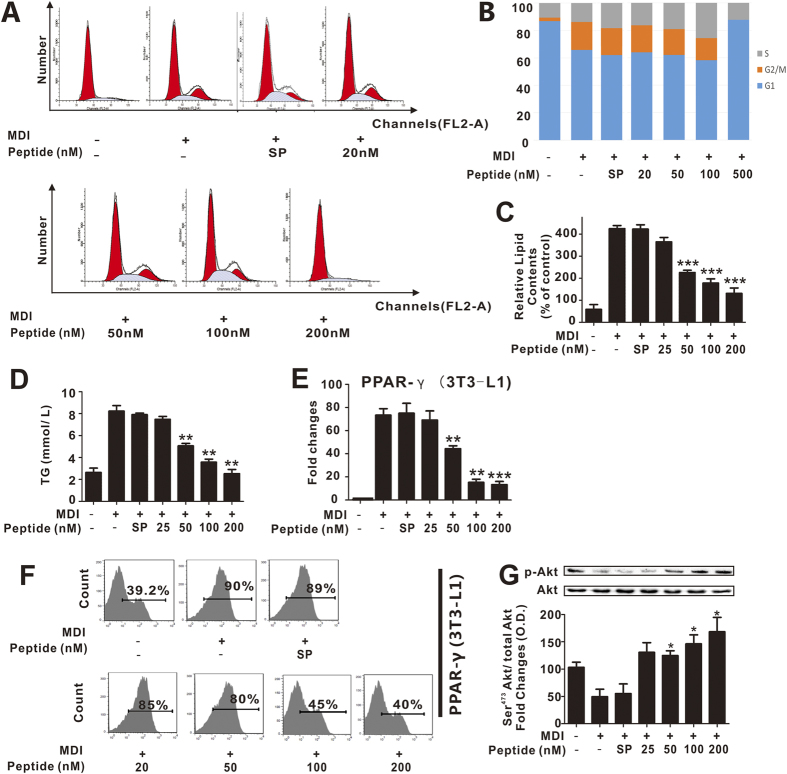
pHBSP suppressed adipogenesis in 3T3-L1 cells. Undifferentiated cells were induced to differentiate into mature 3T3-L1 pre-adipocytes by MDI treatment for 24 hours followed by insulin supplementation. At 24 hours, a subset of cells was used to test MCE, and the remaining cells were used at day 8 to test cell differentiation and expression of PPARγ mRNA. (**A,B)** Effects of pHBSP on MDI-induced cell cycle progression in 3T3-L1 preadipocytes. (**C)** pHBSP inhibited lipid storage, as shown by staining with oil red O solution. (**D)** pHBSP decreased the storage of triglyceride in 3T3-L1 cells. (**E,F**) pHBSP diminished the mRNA (**E**) and protein (**F**) levels of PPARγ. (**G)** pHBSP increased the phosphorylation of Akt in 3T3-L1 cells. N = 6 per group; TG indicates triglycerides, and SP indicates scrambled peptide; data are the mean ± SEM; error bars indicate s.e.m., and significance is indicated by *p < 0.05, **p < 0.01 and ***p < 0.005, as determined by one-way analysis of variance followed by Dunnett’s multiple comparison test.

Mitotic clonal expansion (MCE) is a synchronous process involved in adipogenesis. 3T3-L1 preadipocytes synchronously reenter the cell cycle and undergo MCE after differentiation^35^. Therefore, we first examined whether pHBSP can inhibit MCE by suppressing cell cycle progression in 3T3-L1 preadipocytes. As shown in Fig. 1A,B, undifferentiated 3T3-L1 cells did not undergo cell cycle progression until 8 days following treatment with isobutylmethylxanthine, dexamethasone and insulin (MDI), and pHBSP intervention dose-dependently blocked the progression of 3T3-L1 preadipocytes at the G2 phase, suggesting that pHBSP can suppress 3T3-L1 preadipocyte cell cycle progression. Moreover, the suppression of preadipocyte cell cycle progression may affect adipocyte differentiation. Therefore, we used an oil red O (ORO) staining experiment to detect adipocyte differentiation and found that MDI-mediated lipid and triglyceride accumulation in 3T3-L1 cells were dose-dependently reduced by pHBSP (Fig. 1C, p < 0.005 for doses 200, 100 and 50 nM; Fig. 1D, p < 0.01 for doses 200, 100 and 50 nM), indicating that pHBSP suppressed adipocyte differentiation.

Increased glucose metabolism is crucial for lipid and triglyceride accumulation. Therefore, we detected the activation of Akt, a key molecule that regulates the translocation of GLUT4 and further increases glucose uptake^36^. As shown in Fig. 1G, pHBSP dose-dependently up-regulated Akt phosphorylation, suggesting that pHBSP may increase glucose intake in 3T3-L1 cells (p < 0.05).

PPARγ is a master regulator of adipogenesis in adipocytes; therefore, we further monitored the effect of pHBSP on PPARγ expression in adipocytes. As shown in Fig. 1E,F, the mRNA and protein levels of PPARγ in 3T3-L1 preadipocytes were attenuated by pHBSP in a dose-dependent manner (p < 0.01).

**Figure 2:**
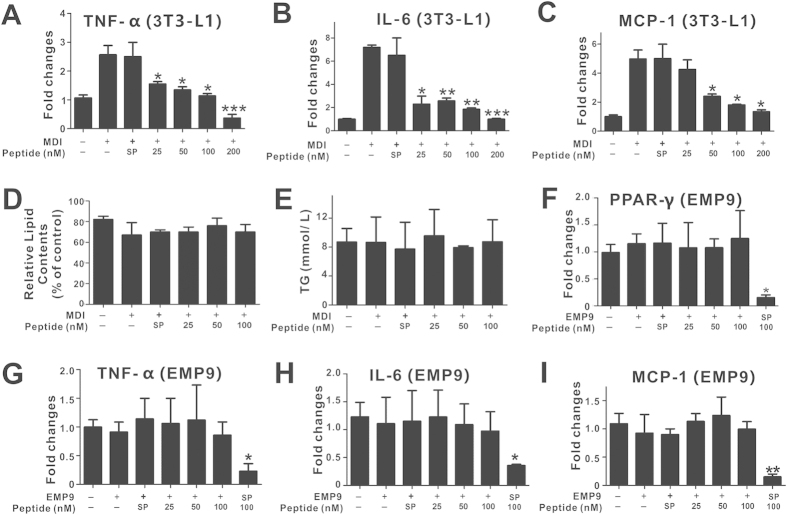
pHBSP suppressed adipokine expression in 3T3-L1 cells. Undifferentiated cells were induced to differentiate into mature 3T3-L1 pre-adipocytes by treating with MDI for 24 hours, and cells at day 8 were used to test the expression of adipokine mRNA. (**A–C)** pHBSP diminished the expression of TNF-α (**A**), IL-6 (**B**) and MCP-1 (**C**) mRNA in 3T3-L1 cells. (**D–E)** Administration of 0.5 mg/mL EMP9 counteracted the effects of pHBSP on differentiation of (**D**) and triglyceride accumulation (**E**) in 3T3-L1 preadipocytes. **(F–I**) Administration of 0.5 mg/mL EMP9 counteracted the effects of pHBSP on PPARγ (**F**), TNF-α (**G**), IL-6 (**H**) and MCP-1 (**I**) expression in 3T3-L1 cells. N = 6 per group; TG indicates triglycerides, and SP indicates scrambled peptide; data are the mean ± SEM; the error bars indicate s.e.m., and significance is indicated by *p < 0.05, **p < 0.01 and ***p < 0.005, as determined by one-way analysis of variance followed by Dunnett’s multiple comparison test.

Because a series of IR-related, pro-inflammatory adipokines are synthesized during adipocyte maturation, we further investigated the effects of pHBSP on the expression levels of select adipokines in 3T3-L1 preadipocytes and found that pHBSP dose-dependently down-regulated the mRNA levels of TNF-α, IL-6 and MCP-1 in MDI-induced 3T3-L1 preadipocytes (Fig. 2A–C, p < 0.05).

To determine whether pHBSP functions through TPR signaling in adipocytes, a reported EPOR antagonist, EPO mimetic peptide-9 (EMP9), was used^37^. Based on the results of a dose-response assay, 0.5 mg/mL EMP9 was used for the following experiments (Supplementary Figure 2). Co-treatment with EMP9 abolished the anti-adipogenesis, anti-adipokine and PPARγ-suppressive effects of pHBSP in 3T3-L1 cells (Fig. 2D–I, p < 0.05), indicating a TPR-dependent effect of pHBSP in 3T3-L1 cells.

### pHBSP inhibits pro-inflammatory cytokine expression in macrophages through TPR

**Figure 3:**
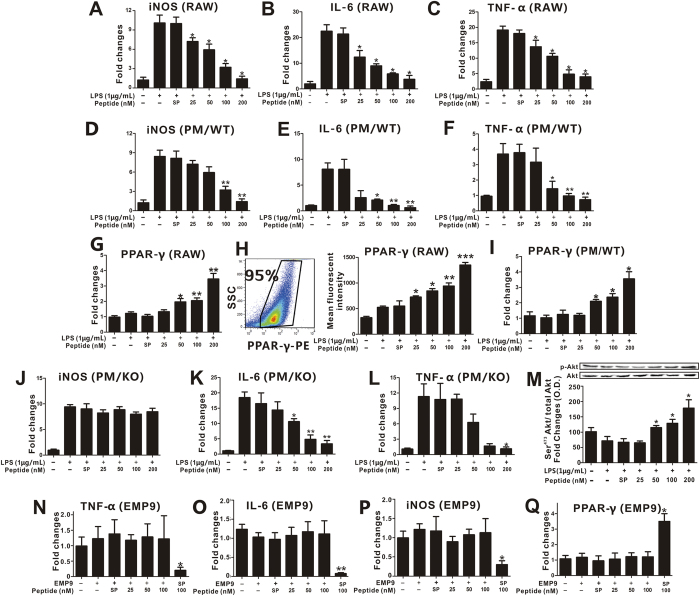
pHBSP inhibits pro-inflammatory cytokine expression in macrophages *in vitro*. (**A–F**) RAW 264.7 cells and peritoneal macrophages were treated with 1 μg/mL LPS followed by various doses of pHBSP or PBS/scrambled peptide for 4 hours. pHBSP diminished the expression of iNOS (**A**), IL-6 (**B**) and TNF-α (**C**) mRNA in RAW264.7 cells and the expression of iNOS (**D**), IL-6 (**E**) and TNF-α (**F**) mRNA in peritoneal macrophages. (**G–I)** LPS-treated RAW 264.7 cells and peritoneal macrophages were subjected to various doses of pHBSP or PBS/scrambled peptide control and then the mRNA (**G**) and protein (**H**) levels of PPARγ in RAW264.7 cells and the mRNA levels of PPARγ (**I**) in peritoneal macrophages were measured by RT-PCR and flow cytometry, respectively. (**J–L)** Peritoneal macrophages were isolated from Mac-PPARγ knockout mice and were treated with 1 μg/mL LPS followed by various doses of pHBSP or PBS/scrambled peptide for 4 hours. The expression of iNOS (**J**), IL-6 (**K**) and TNF-α (**L**) mRNA in PPARγ knockout macrophages was analyzed. (**M**) pHBSP increased Akt phosphorylation in LPS-treated RAW 264.7 cells. (**N–Q**) Administration of 0.5 mg/mL EMP9 counteracted the effects of pHBSP on the expression of TNF-α (**M**), IL-6 (**N**), iNOS (**O**) and PPARγ (**P**) mRNA in RAW264.7 cells. N = 3 per group; PM indicates peritoneal macrophages; SP indicates scrambled peptide; data are the mean ± SEM; error bars indicate s.e.m., and significance is indicated by *p < 0.05, **p < 0.01 and ***p < 0.005, as determined by one-way analysis of variance followed by Dunnett’s multiple comparison test.

Subsequently, we tested the effects of pHBSP on the inflammatory activation of macrophages. As shown in Fig. 3A–F, pHBSP dose-dependently suppressed LPS-induced increases in iNOS, IL-6 and TNF-α mRNA levels in RAW264.7 macrophages and peritoneal macrophages. Interestingly, in contrast to adipocytes, in RAW264.7 macrophages, pHBSP dose-dependently increased the mRNA and protein levels of PPARγ (Fig. 3G,H, p < 0.05). Furthermore, pHBSP dose-dependently induced PPARγ mRNA levels in peritoneal macrophages (Fig. 3I, p < 0.05). In macrophages, the nuclear receptor PPARγ modulates cytokine expression; therefore, we next explored whether pHBSP exerted its anti-inflammatory function via PPARγ in macrophages. We found that in PPARγ-knockdown peritoneal macrophages, the effect of pHBSP on iNOS expression was abolished; however, the effects of pHBSP on TNF-α and IL-6 expression remained unchanged (Fig. 3J–L, p < 0.05), indicating that the suppressive effect of pHBSP on macrophage inflammatory activation was partly due to its promotion of PPARγ expression.

**Figure 4:**
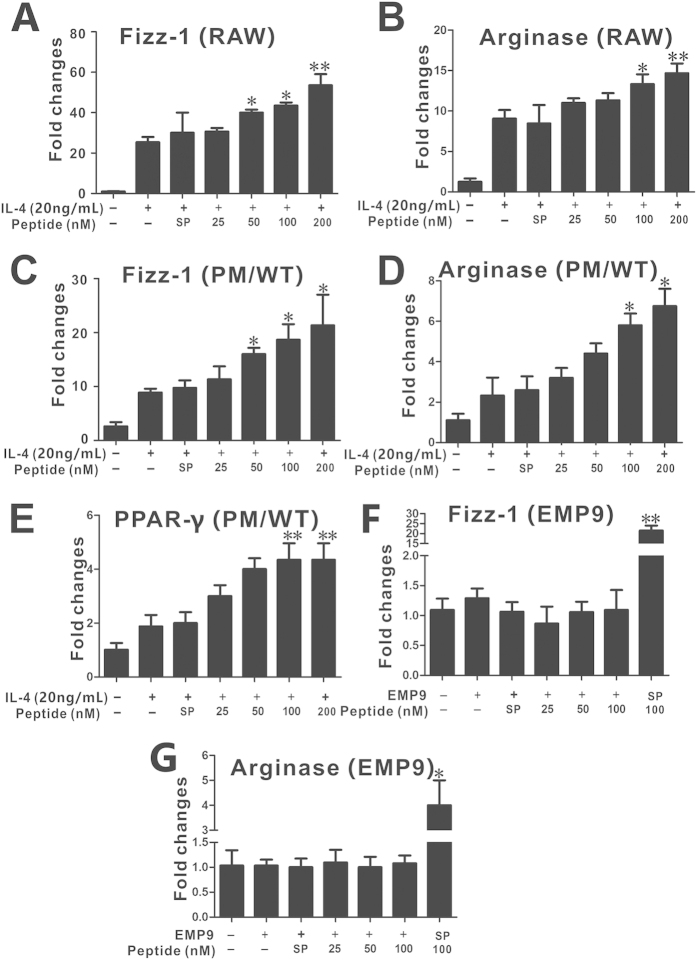
pHBSP promotes M2 marker expression *in vitro*. (**A–E**) RAW 264.7 cells and peritoneal macrophages were treated with 20 ng/mL IL-4 followed by various doses of pHBSP or PBS/scrambled peptide control for 24 hours. pHBSP increased the expression of Fizz-1 (**A**) and arginase-1 (**B**) mRNA in RAW264.7 cells and the expression of Fizz-1 (**C**), arginase-1 (**D**) and PP**A**Rγ (**E**) mRNA in peritoneal macrophages. (**F,G**) Administration of 0.5 mg/mL EMP9 counteracted the effects of pHBSP on the expression of Fizz**-**1 (**F**) and arginase-1 (**G**) mRNA in RAW264.7 cells. N = 3 per group; PM indicates peritoneal macrophages; SP indicates scrambled peptide; data are the mean ± SEM; the error bars indicate s.e.m., and significance is indicated by *p < 0.05 and **p < 0.01, as determined by one-way analysis of variance followed by Dunnett’s multiple comparison test.

Because Akt activation is involved in the activation of macrophages, we next evaluated Akt phosphorylation in macrophages following pHBSP stimulation. As shown in Fig. 4M, pHBSP dose-dependently increased Akt phosphorylation (p < 0.05).

In addition, we investigated whether pHBSP inhibits macrophage inflammatory activation through TPR. As shown in Fig. 3N–Q, EMP9 deprived the anti-inflammatory and PPARγ-inducing functions of pHBSP in macrophages (p < 0.05).

The down-regulation of pro-inflammatory cytokine expression implicates that pHBSP may tether the M1 polarization in macrophages; therefore, we further analyzed whether pHBSP can promote M2 polarization in macrophages. We found that co-treatment with pHBSP increased the expression of Fizz-1, Arginase-1 and PPARγ compared to treatment with IL-4 alone (Fig. 4A–E, p < 0.05), which was deprived by EMP9 (Fig. 4F–G, p < 0.05).

### pHBSP attenuates high-fat diet (HFD)-induced obesity and IR without stimulating hematopoiesis

**Figure 5:**
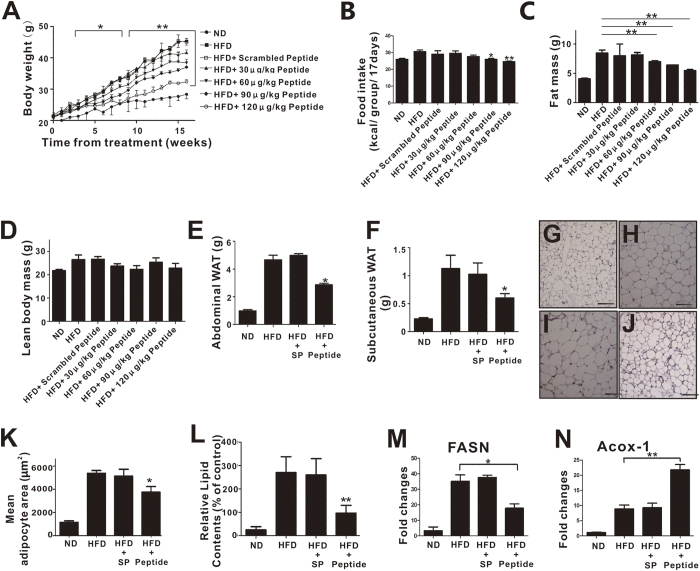
pHBSP improves obesity. C57BL/6 male mice (5–6 weeks of age) were put on an HFD and received exogenous pHBSP at different doses (30, 60, 90, and 120 μg/kg). pHBSP was injected intraperitoneally using insulin syringes every other day. A subset of HFD-fed mice were injected with PBS/scrambled peptide only to serve as sham controls. (**A–F**) Body weight (**A**) and food intake (**B**) were measured once a week, and fat mass (**C**), lean mass (**D**), abdominal adipose (including mesenteric, retroperitoneal, and gonadal adipose) weight (**E**) and subcutaneous adipose weight (**F**) were measured when the mice were sacrificed at week 16. (**G–K**) Paratesticular WAT for H.E. staining was also performed after the mice were sacrificed at week 16. (**G–J**) shows representative H.E. staining of epididymal fat pad sections from ND-fed mice (**G**), HFD-fed mice (**H**), scrambled peptide-treated HFD-fed mice (**I**) or 120 μg/kg pHBSP-treated HFD-fed mice (**J**). Bar graph shows the mean adipocyte area (μm^2^) (**K**). (**L–N)** 120 μg/kg pHBSP decreased lipid storage in adipose tissue (**L**), decreased the expression of FASN (**M**) in WAT and increased Acox-1 expression in muscle (**N**). N = 6 per group; SP indicates scrambled peptide; Peptide indicates 120 μg/kg pHBSP; data are the mean ± SEM; the error bars indicate s.e.m., and significance is indicated by *p < 0.05 and **p < 0.01, as determined by Student’s t-test. The scale bars are 100 μm.

Due to the potent anti-adipogenic and anti-inflammatory effects of pHBSP *in vitro*, we next explored pHBSP’s effects on obesity and IR *in vivo*. HFD was used to induce obesity in mice by administering varying doses of pHBSP (120, 90, 60 and 30 μg/kg) every other day from the beginning of HFD administration. At week 16, we found that HFD induced greater obesity (45.33 ± 1.04 g) than normal diet in mice (28.42 ± 1.45 g, Fig. 5A, p < 0.05). Indeed, pHBSP dose-dependently reduced body weight in mice with HFD-induced obesity (Fig. 5A, p < 0.05); food intake was also decreased by pHBSP in mice with diet-induced obesity (DIO) (Fig. 5B, p<0.05). Furthermore, pHBSP decreased fat mass in DIO mice by up to 3.36 ± 0.42 g (Fig. 5C, p < 0.01) but did not cause significant changes in lean body mass (Fig. 5D, P > 0.05). Next, quantitative analysis of fat-pad weight disclosed that pHBSP treatment significantly decreased abdominal (49.34%, Fig. 5E, p < 0.05) and subcutaneous adipose weight (52.65%, Fig. 5F, p < 0.05) in DIO mice. Moreover, histological examination of epididymal white adipose tissue (WAT) revealed that the administration of pHBSP significantly reduced adipocyte size compared with that in a vehicle control group (Fig. 5G–K, p < 0.05). Moreover, ORO staining showed that pHBSP diminished lipid storage in adipose tissue (Fig. 5L, p < 0.01).

Because fat mass gain is closely related to fatty acid oxidation, we examined the effects of pHBSP on fatty acid synthase gene (FASN) expression in WAT and Acox-1 expression in muscle and found that pHBSP decreased FASN (Fig. 5M, p < 0.05) expression, whereas it increased Acox-1 (Fig. 5N, p < 0.05) expression.

**Figure 6:**
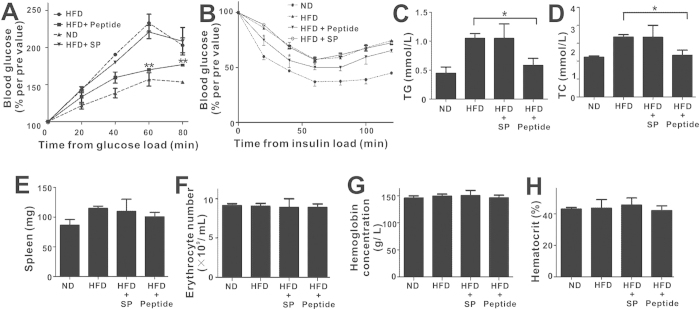
pHBSP improves insulin resistance but does not stimulate hematopoiesis. C57BL/6 male mice (5–6 weeks of age) were put on an HFD and received exogenous pHBSP at different doses (30, 60, 90, and 120 μg/kg). pHBSP was injected intraperitoneally using insulin syringes every other day. A subset of HFD-fed mice were injected with PBS/scrambled peptide to serve as a sham control. (**A,B)** Glucose and insulin tolerance tests were performed at week 16 on ND-fed mice, HFD-fed mice, scrambled peptide-treated HFD-fed mice and 120 **μ**g/kg peptide-treated HFD-fed mice. (**C,D)** At week 16, the mice in each group were sacrificed to take peripheral blood for detection of blood triglyceride and total cholesterol levels. Treatment with 120 μg/kg pHBSP attenuated circulating triglyceride (**C**) and total cholesterol (**D**) levels in HFD-fed mice. (**E–H**) At week 16, the mice were sacrificed for spleen and peripheral blood harvest for the detection of erythropoietic activation. (**E–H**) show spleen weight measurements (**E**), peripheral red blood cell numbers (**F**), hemoglobin levels (**G**) and hematocrit percentage (**H**) in each group. N = 6 per group; SP indicates scrambled peptide; Peptide indicates 120 μg/kg pHBSP; data are the mean ± SEM; the error bars indicate s.e.m., and significance is indicated by *p < 0.05 and **p < 0.01.

Because IR is an essential event towards metabolic dysregulation in obesity, we tested the levels of glucose and insulin tolerance following pHBSP treatment in DIO mice. pHBSP treatment normalized both glucose disposal rate (Fig. 6A, p < 0.05) and insulin sensitivity (Fig. 6B, p < 0.05). Furthermore, circulating triglyceride and total cholesterol levels in DIO mice were markedly attenuated by pHBSP (Fig. 6C,D, p < 0.05).

In addition, we investigated whether pHBSP possessed hematopoietic properties during obesity treatment. Administration of pHBSP to HFD-fed mice every other day for 16 weeks did not alter spleen weight (Fig. 6E), the number of peripheral red blood cells (Fig. 6F), hemoglobin level (Fig. 6G) or hematocrit percentage (Fig. 6H), suggesting that pHBSP did not stimulate hematopoiesis during obesity treatment.

### pHBSP attenuates obesity-mediated inflammation and macrophage accumulation

**Figure 7:**
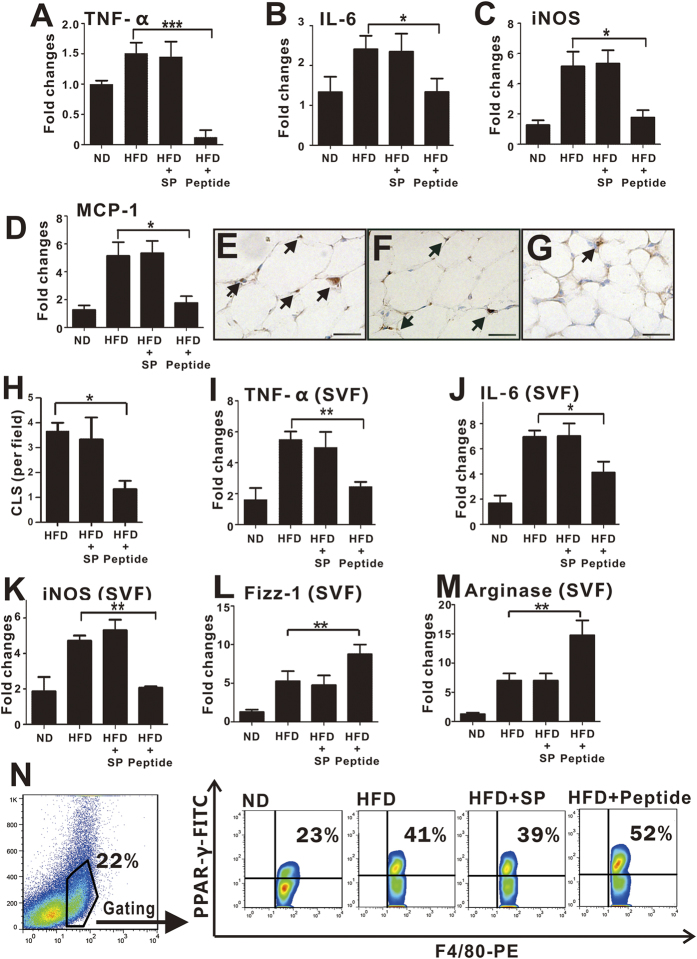
pHBSP attenuates inflammation *in vivo*. (**A–D**) PBS, scrambled peptide or pHBSP (120 μg/kg/2days) was given to HFD-fed mice, and ND-fed mice were injected with PBS as a sham control. At week 16, the mice were sacrificed to take WAT for RT-PCR. (**A–D)** The expression of TNF-α (**A**), IL-6 (**B**), iNOS (**C**) and MCP-1 (**D**) in WAT decreased following 120 μg/kg pHBSP treatment compared with the control group. (**E–H**) F4/80 staining was applied to visualize ATM infiltration in the adipose tissue taken at week 16. The arrows indicate CLS formation in adipose tissue in HFD-fed mice treated with PBS, 120 μg/kg scrambled peptide or 120 μg/kg pHBSP. ATM (F4/80^+^) infiltration was significantly suppressed by 120 μg/kg pHBSP treatment compared to PBS and scrambled peptide control treatment (n = 6). (**I–M**) SVF cells were isolated from adipose tissue at week 16, and 120 μg/kg pHBSP diminished the expression of TNF-α (**I**), IL-6 (**J**) and iNOS (**K**) mRNA in SVF cells, whereas the treatment up-regulated the expression of Fizz-1 (**L**) and arginase-1 (**M**) mRNA. (**N**) The mice were sacrificed at week 16 to harvest SVF cells from adipose tissue, and the protein levels of PPARγ in F4/80^+^ SVF cells were measured. Treatment with 120 μg/kg pHBSP increased PPARγ expression in F4/80^+^ SVF. N = 6 per group; SVF indicates stromal cular fraction; SP indicates scrambled peptide; Peptide indicates 120 μg/kg pHBSP; data are the mean ± SEM; the error bars indicate s.e.m., and significance is indicated by *p < 0.05, **p < 0.01 and ***p < 0.005, as determined by one-way analysis of variance followed by Dunnett’s multiple comparison test. The scale bars are 50 μm.

Given the important contribution of local inflammation to the development of IR in adipose tissue, we next examined the expression of pro-inflammatory cytokines in epididymal WAT of HFD-fed mice and found that levels of TNF-α, IL-6, iNOS and MCP-1 were greatly reduced following pHBSP treatment for 16 weeks (Fig. 7A–D, p < 0.05).

Because macrophages play dominant roles in obesity-associated inflammation, we next examined whether pHBSP affected ATMs in HFD-fed mice. First, we found that pHBSP treatment dramatically reduced the accumulation of F4/80^+^ macrophage clusters (CLSs) in epididymal fat pads (Fig. 7E–H). Then, we measured the expression of certain cytokines in ATMs following pHBSP treatment. Epididymal fat pad stromal vascular cells (SVF) were isolated, and the mRNA levels of different cytokines were analyzed. pHBSP administration decreased levels of TNF-α, IL-6 and iNOS, whereas it increased the expression of M2 macrophage-related molecules, such as Fizz-1 and Arginase-1, in the SVF of HFD-fed mice (Fig. 7I–M, p < 0.01). Furthermore, pHBSP significantly increased PPARγ expression in F4/80^+^ ATMs from the SVF of HFD-fed mice (Fig. 7N, p < 0.05).

## Discussion

Growing attention has focused on the nonerythropoietic activity of EPO, such as its role in obesity regulation^38^. It has been reported that EPO reduces food intake and preadipocyte differentiation and increases fat oxidation, leading to improved fat mass and IR in DIO mice^24^,^39^,^40^,^41^. However, safety concerns have been raised over long-term and high-dose application of EPO, which has been associated with hypertension and thrombosis^25^. The EPO analogue pHBSP was reported to retain the tissue-protective functions of EPO but without its erythropoietic properties^26^. Specifically, Collino *et al.* reported that chronic treatment with pHBSP improved metabolic abnormalities induced by HFD^42^. Furthermore, in the clinic, pHBSP was demonstrated to improve metabolic indexes in patients suffering from type 2 diabetes^43^. Although these studies are exciting and provide the first strong evidence to support further investigations of pHBSP effects on obesity and related disorders, several important issues have been left unanswered from these studies, such as whether pHBSP can effectively prevent the development of obesity, what the dose-response of pHBSP is in obese individuals and, more importantly, whether other mechanisms exist regarding the effects of pHBSP on obesity modulation. Our current results show that preventive treatment with pHBSP significantly reduced weight gain and improved IR, suggesting a preventive effect of pHBSP in obesity modulation. Moreover, pHBSP was observed to have a dose-dependent effect, ranging from 30 to 120 μg/kg, in obesity modulation, providing evidence for a wider dose choice. Mechanistically, unlike Collino’s research, which mainly focused on skeletal muscle, our research revealed the anti-inflammatory and anti-adipogenesis effects of pHBSP peptide on adipocytes and macrophages, suggesting that this peptide may improve obesity through multiple mechanisms and target cells. Therefore, together with Collino’s report, our current investigations prove that pHBSP has protective effects in obesity modulation and support the application of PHBSP in treating obesity.

Obesity is associated with hyperplasia and hypertrophy of adipocytes in white adipose tissue, the main site for excess energy storage^44^. Increased adipose tissue mass may result from increased sizes and numbers of adipocytes, which principally reflects the amount of stored triglycerides^14^. Therefore, reducing adipocyte hyperplasia and/or hypertrophy would of benefit in obesity treatment. In this study, mice fed an HFD suffered from increased fat mass, higher levels of blood triglycerides and expanded adipocytes. Notably, pHBSP intervention significantly reduced food intake, fat mass, adipocyte size, and triglyceride synthesis/storage in both adipose tissue and adipocytes. Furthermore, pHBSP blocked 3T3-L1 preadipocyte MCE. These data are consistent with previous studies documenting the effect of EPO on obesity^45^. In addition, we discovered that pHBSP can down-regulate FASN in WAT while promoting Acox-1 expression in muscle. FASN is a well-known rate-limiting enzyme in fatty acid synthesis, and in addition to being rate-liming Acox-1 is the first enzyme in the fatty acid beta-oxidation pathway^46^. This suggests that pHBSP can inhibit fatty acid synthesis and promote fatty acid oxidation, which may be another potential mechanism behind pHBSP’s suppression of body weight and fat accumulation in obesity.

Obesity increases the risk for T2D by inducing IR. Although the manner in which obesity influences IR is not fully known, over the past several years, the significant contributions of pro-inflammatory molecules, such as TNF-α, IL-6, iNOS and MCP-1, to IR have been elucidated^14^. The deletion of TNF-α has been found to ameliorate inflammation and IR in DIO, and treatment with antibodies against TNF-α can increase insulin sensitivity in muscle^47^,^48^. IL-6 can reduce insulin-dependent hepatic glycogen synthesis and glucose uptake in adipocytes, whereas it enhances insulin-dependent glycogen synthesis and glucose uptake in myotubes^49^. MCP-1 contributes to macrophage infiltration in adipose tissue^50^. Macrophages and adipocytes are the main source of these molecules in obesity. In the current study, pHBSP ameliorated IR in obese mice and, notably, suppressed the expression of TNF-α, IL-6, iNOS and MCP-1 in both adipocytes and macrophages, indicating that pHBSP may improve IR through suppressing the inflammatory activation of macrophages and adipocytes.

Mechanistically, our study revealed that pHBSP significantly inhibited PPARγ expression in 3T3-L1 preadipocytes. PPARγ is a master regulator of adipogenesis, and down-regulation of PPARγ in adipocytes decreases fat mass^51^. Moreover, PPARγ knockout mice completely lack adipose tissue^52^. In addition, adipocyte differentiation is dependent on PPARγ, and the activation of PPARγ results in increased gene expression of adipogenic markers, such as fatty acid synthase^51^. Moreover, PPARγ also affects the expression of IR-related adipokines, such as TNF-α, IL-6 and MCP-1, in adipocytes^53^. Therefore, given the important roles that PPARγ plays in adipocytes, it is reasonable to speculate that pHBSP may at least partly inhibit adipogenesis and inflammatory cytokine expression in adipocytes through the down-regulation of PPARγ expression.

Conversely, we discovered that pHBSP induced PPARγ expression in macrophages. Accumulated data have shown that PPARγ is a ligand-dependent nuclear receptor, which has potentially anti-inflammatory effects in macrophages. The macrophage-specific deletion of PPARγ leads to an increase in local inflammation in adipose tissue, characterized by decreased expression of TNF-α, IL-6 and iNOS. On the contrary, the PPARγ agonist TZD can inhibit the production of inflammatory cytokines in macrophages^54^,^55^,^56^. Using PPARγ knockout macrophages, we found that pHBSP reduced iNOS expression, but not TNF-α and IL-6 expression, via PPARγ, indicating the existence of other signaling pathways for pHBSP-related anti-inflammatory effects. Previous investigations have shown that EPO inhibits the expression of TNF-α and IL-6 by suppressing Nuclear Factor-κB (NFκB) activation in macrophages^57^, suggesting that the EPO-derived pHBSP might inhibit NFκB activation as well. Therefore, these data suggest that pHBSP may suppress inflammatory macrophage activation through multiple signaling pathways, leading to increased PPARγ expression and decreased NFκB activation.

Although the manner in which PPARγ is differently regulated by pHBSP in adipocytes and macrophages remains elusive, previous EPO studies may provide some clues. It has been reported that EPO activates ERK, which further increased PPARγ phosphorylation and decreased PPARγ activity in 3T3-L1 cells^58^. However, in macrophages, the activation of the ERK pathway by EPO increased the expression of C/EBPβ, a transcriptional activator for PPARγ^59^,^60^, leading to the up-regulation of PPARγ. Therefore, pHBSP may modulate PPARγ expression differently through different signal pathways in adipocytes and macrophages.

In summary, the protective role of pHBSP in obesity and IR was established in the current study. pHBSP may be a promising candidate for DIO intervention, improving IR and attenuating inflammation through multiple mechanisms.

## Methods

All animal care and experiments were approved by the Animal Care and Use Committees of Third Military Medical University. All methods were carried out in accordance with the approved guidelines.

### 3T3-L1 cell culture and adipocyte differentiation

For 3T3-L1 cell culture, 3T3-L1 preadipocytes were first grown in complete DMEM (Gibco, Grand Island, NY, USA) with 10% bovine calf serum at 37 °C and 5% CO_2_ until 3 days post-confluence. Next, the confluent cells were incubated in complete DMEM containing 10% FBS (Gibco, Grand Island, NY, USA), 1 μM dexamethasone, 0.5 mM isobutylmethylxanthine and 5 μg/mL insulin at 37 °C and 5% CO_2_ for 2 days. Then, the medium was replaced with complete DMEM with 10% FBS and 5 μg/mL insulin for another 2 days. Finally, the medium was replaced with complete DMEM containing 10% FBS (with different doses of pHBSP or PBS/scrambled peptide control), with the medium being replaced every 2 days.

In order to keep the comparability of the whole study, we set both PBS and scrambled peptide as control.

### ORO staining

Mature adipocytes were fixed with 10% formalin for 1 minute. Then, the formalin was removed, and the cells were stained with ORO solution (Sigma-Aldrich) and washed with PBS. For quantification, the ORO was re-suspended in isopropyl alcohol, and the absorbance was measured spectrophotometrically at 515 nm.

### Isolation of mouse peritoneal macrophages, and cell model establishment, culture and treatment

To isolate mouse peritoneal macrophages, a peritoneal lavage with 20 mL DMEM containing 2% FBS and 100 units/mL penicillin was applied. After centrifugation (1000 rpm, 5 min), red blood cells were lysed in Red Blood Cell Lysis Buffer (Biolegend, San Diego, CA, USA), and mononuclear cells were plated and maintained in complete DMEM containing 10% FBS, 100 units/mL penicillin and 100 units/mL streptomycin at 37 °C for 2 h. Then, non-adherent cells were removed, and the remaining adherent cells were cultured in complete DMEM with 10% FBS.

For RAW264.7 cell culture, the cells were grown in complete DMEM with 10% fetal calf serum, 100 units/mL penicillin and 100 units/mL streptomycin at 37 °C and 5% CO_2_.

For the M1/M2 model, macrophages were induced with 1 μg/mL LPS for 8 h or 20 ng/mL IL-4 for 24 h, together with different doses of pHBSP (synthetized by Chinesepeptide Corporation, Hangzhou, China, sequence: QEQLERALNSS; purity: 98%) or PBS/200 nM scrambled peptide.

It has been reported that TPR consists of EPOR and βcR subunits^42^. To inhibit TPR function, erythropoietin mimetic peptide-9 (EMP-9), a proven EPOR antagonist, was applied. The EMP-9 was dissolved in complete DMEM to a final concentration of 0.5 mg/mL.

### Animals and pHBSP intervention

Male C57BL/6 mice (5–6 weeks old, Vital River, Beijing, China) were fed either a standard maintenance diet (normal chow, 2844 kcal/kg, 4% crude fat, Daping Hospital, Chongqing, China) or an HFD containing 5240 kcal/kg (high fat, 34.9% crude fat, Daping Hospital, Chongqing, China). All animals in this study were subject to controlled temperature (22 ± 1 °C) and lighting (lights on 06:00 to 18:00) conditions. All animal experimental procedures were subject to approval by the Animal Care and Use Committees of Third Military Medical University. All efforts were made to minimize the number of animals used and their suffering.

The mice were separated randomly into distinct groups based on pHBSP dose (n = 6 mice/group, doses were 30, 60, 90, and 120 μg/kg); the doses of pHBSP were based on our former study, which showed the therapeutic effect of the long-term use of pHBSP on experimental autoimmune neuritis^33^. The interval of pHBSP administration was based on previous clinical data that demonstrated that the repetitive administration of pHBSP was effective despite its short half-life^34^. The pHBSP was dissolved in PBS. An equivalent volume of PBS and 120 μg/kg scrambled peptide was used as a control group. The PBS and peptide mixture were administered intraperitoneally using insulin syringes (B. Braun Melsungen AG, Germany) every other day.

### Body weight and fat mass measurement

The mice were individually weighed weekly. At week 16, they were sacrificed by cervical dislocation. Next, subcutaneous fat deposits were dissected and weighted. Then, abdominal fat deposits (including fat pads associated with the epididymis, kidneys and mesentery) were isolated and weighted. Together, the subcutaneous and abdominal fat mass constituted the fat mass, and the body mass minus the cleared out fat mass constituted the lean body mass.

### Glucose and insulin tolerance tests

Mice (n = 6/group) were fasted for 16 hours and challenged with 1) an intraperitoneal load of glucose (1 g/kg) for glucose tolerance testing, or 2) an intraperitoneal load of human insulin (0.375 IU/kg, Actrapid, Novo Nordic, Denmark) for insulin tolerance testing. Blood samples (10 μl) were taken retro-orbitally from conscious mice at 0, 30, 60, 90, 120, and 180 min after glucose or insulin load. Blood glucose levels were determined with the help of a Onetouch Ultra (Johnson & Johnson, USA).

### Epididymal fat pad SVF isolation and ATM purification

First, epididymal fat pads from each group were collected and collagenase (Sigma-Aldrich, 1 mg/mL) was added and incubated at 37 °C for 80 minutes with shaking. Next, the resultant cell suspensions were filtered through 70-μm filters. After centrifugation (300 *g*, 5 min), floating mature adipocytes were removed. Then, the cells were plated and cultured in complete DMEM containing 10% FBS, 100 units/mL penicillin and 100 units/mL streptomycin at 37 °C for 2 h. Following this, non-adherent cells were removed, and the remaining adherent cells were analyzed.

### Flow cytometry

Single-cell suspensions from SVF, 3T3-L1 cells, and macrophages were washed twice in staining buffer, re-suspended, and incubated with labeled antibodies at 4 °C for 30 min. After that, the cells were washed twice with staining buffer and analyzed. F4/80 and PPARγ staining was performed as per manufacturer’s instructions (Sungene Biotech, Tianjin, China).

For cell cycle analysis, two days post-confluence, 3T3-L1 preadipocytes were induced to differentiate with different doses of pHBSP or PBS/scrambled peptide. Twenty-four hours later, the cells were harvested and fixed with 75% ethanol for 12 h at 4 °C. Then, the ethanol was removed, and the cells were washed twice with PBS and stained with propidium iodide solution containing 20 μg/mL RNase for 30 min.

Flow cytometry analysis was performed on a Becton–Dickinson FACScan system, and the data were analyzed using FlowJo software (version 7.6.1, Tree Star Software, San Carlos, CA, USA).

### Western blot analysis

For western blotting, the cells were washed twice with ice-cold PBS and lysed with cell lysis buffer (Beyotime, China) according to the manufacturer’s protocol. Protein lysates were separated via 12% SDS-polyacrylamide gel electrophoresis for 1.5 h and transferred to a polyvinylidene difluoride membrane for 1 h. Then, the membrane was blocked with 5% non-fat milk for 1 h at 25 °C and incubated with primary antibodies against Akt and p-Akt (1:400, Santa Cruz, Texas, USA) at 4 °C overnight, followed by incubation with secondary antibodies (1:2000, Thermo Fisher, Waltham, MA, USA) conjugated to horseradish peroxidase for 1 h at 25 °C. Detection was performed using an ECL chemiluminescence kit (Millipore, Billerica, MA, USA), and the optical densities of phosphoprotein/total protein were analyzed using MyImage Analysis software (Thermo Fisher, Waltham, MA, USA).

### Tissue preparation, RNA isolation, reverse transcription and real-time PCR

At week 16, the mice (n = 6/group) were perfused intracardially with 4 °C PBS under anesthesia, after which WAT were quickly removed and stored in liquid nitrogen until RNA isolation. Total RNA was isolated using Trizol LS reagent (Invitrogen, Carlsbad, CA, USA) and reverse transcribed into cDNA using a Quantscript RT Kit (TIANGEN Biotech, Beijing, China).

The cDNA was used to assess the relative expression of genes using RealMasterMix (SYBR green I) according to the manufacturer’s protocol (TIANGEN Biotech, Beijing, China). Real-time measurements of gene expression were performed with a DNA Engine Opticon 2 Real-Time Cycler PCR detection system (Bio-Rad Lab., Richmond, CA, USA). The primers used to measure gene expression are described in Table 1.

### Histology

To evaluate ATM accumulation, the mice were deeply anesthetized with ether and perfused intracardially with 4% paraformaldehyde at 4 °C in PBS. Epididymal fat pads were quickly removed and post-fixed in 4% paraformaldehyde overnight at 4 °C. The tissues were cut into two equally long segments, embedded in paraffin, serially sectioned (3 μm) and mounted on silan-covered slides.

Routine hematoxylin and eosin (HE) staining was performed as described previously^33^. For immunohistochemistry, after dewaxing, cross-sections were boiled (in a 600 W microwave oven) for 15 min in citrate buffer (2.1 g sodium citrate/L, pH 6). Endogenous peroxidase was inhibited with 1% H_2_O_2_ in methanol for 15 minutes. The sections were incubated with 10% normal pig serum (Biochrom, Berlin, Germany) to block non-specific binding of immunoglobulins and then with an anti-mouse F4/80 antibody (1:50; Abcam, Cambridge, UK) for macrophage identification. Antibody binding to tissue sections was visualized with a biotinylated IgG F(ab)_2_ secondary antibody fragment (DAKO, Hamburg, Germany). Subsequently, the sections were incubated with a horseradish peroxidase-conjugated streptavidin complex (DAKO, Hamburg, Germany) followed by development with diaminobenzidine (DAB) substrate (Fluka, Neu-Ulm, Germany). Finally, the sections were counterstained with Maier’s Hemalum.

To evaluate the immunostaining data, CLSs were identified as adipocytes completely surrounded by F4/80^+^ cells and were quantified in 6 random fields in all animals. Images of WAT cross-sections were collected under 50× magnification using a Nikon Coolscope (Nikon, Düsseldorf, Germany) with fixed parameters. The images were analyzed using MetaMorph Offline 7.1 (Molecular Devices, Toronto, Canada). The results are given as the numbers of CLSs per area in WAT cross-sections and include the standard errors of the means (SEM).

### Data evaluation and statistical analysis

The data are expressed as mean ± s.e.m. Comparisons between two groups were made using two-tailed non-paired Student’s t-test. Statistical differences between three or more groups were evaluated by one-way analysis of variance with Dunnet’s multiple comparison post hoc tests at α = 0.05. Area under the curve assessment was used in the glucose and insulin tolerance tests. A p value of <0.05 was considered to be statistically significant.

## Additional Information

**How to cite this article**: Liu, Y. *et al.* Nonerythropoietic Erythropoietin-Derived Peptide Suppresses Adipogenesis, Inflammation, Obesity and Insulin Resistance. *Sci. Rep.* **5**, 15134; doi: 10.1038/srep15134 (2015).

## Supplementary Material

Supplementary Figure

## Figures and Tables

**Table 1:** Sequences of primers for quantitative RT-PCR.

Pimers	Sequences
FASN forward	GGACATGGTCACAGACGATGAC
FASN reverse	GTCGAACTTGGACAGATCCTTCA
Leptin forward	TCCAGAAAGTCCAGGATGACAC
Leptin reverse	CACATTTTGGGAAGGCAGG
Adiponectin forward	AAGGACAAGGCCGTTCTCT
Adiponectin reverse	TATGGGTAGTTGCAGTCAGTTGG
PPARγ forward	GGAATGGGAGTGGTCATCCA
PPARγ reverse	CCCACCAACTTCGGAATC
TNF-α forward	CGTCAGCCGATTTGCTATCT
TNF-α reverse	CGGACTCCGCAAAGTCTAAG
IL-6 forward	ACAAGTCGGAGGCTTAATTAC
IL-6 reverse	TTGCCATTGCACAACTCTTTC
iNOS forward	AATGGCAACATCAGGTCGGCCATCACT
iNOS reverse	GCTGTGTGTCACAGAAGTCTCGAACTC
MCP-1 forward	CTTCCTCCACCACCATGC
Fizz-1 forward	TCCCAGTGAATACTGATGAGA
Fizz-1 reverse	CCACTCTGGATCTCCCAAGA
Arginase forward	AAGCCTGGTCTGCTGGAAAAA
Arginase reverse	CTGGTTGTCAGGGGAGTGTT
MCP-1 reverse	CCAGCCGGCAACTGTGA
β-Actin forward	ATGGGTCAGAAGGACTCCTACG
β-Actin reverse	AGTGGTACGACCAGAGGCATAC
