# Supplementary material for: Nonerythropoietic Erythropoietin-Derived Peptide Suppresses Adipogenesis, Inflammation, Obesity and Insulin Resistance
Source: Sci Rep. 2015 Oct 13;5:15134. doi: 10.1038/srep15134 (PMC4602313; doi:10.1038/srep15134)
Supplement: Supplementary Figure [file srep15134-s1.pdf]

Supporting information

Nonerythropoietic Erythropoietin-Derived Peptide Suppresses Adipogenesis, Inflammation, Obesity and Insulin Resistance

Rongchen Shi, Yuqi Liu, Zongwei Liu, Jinsong Wang, Bangwei Luo, Wei Liu, Shufeng Wang, Zhiren Zhang

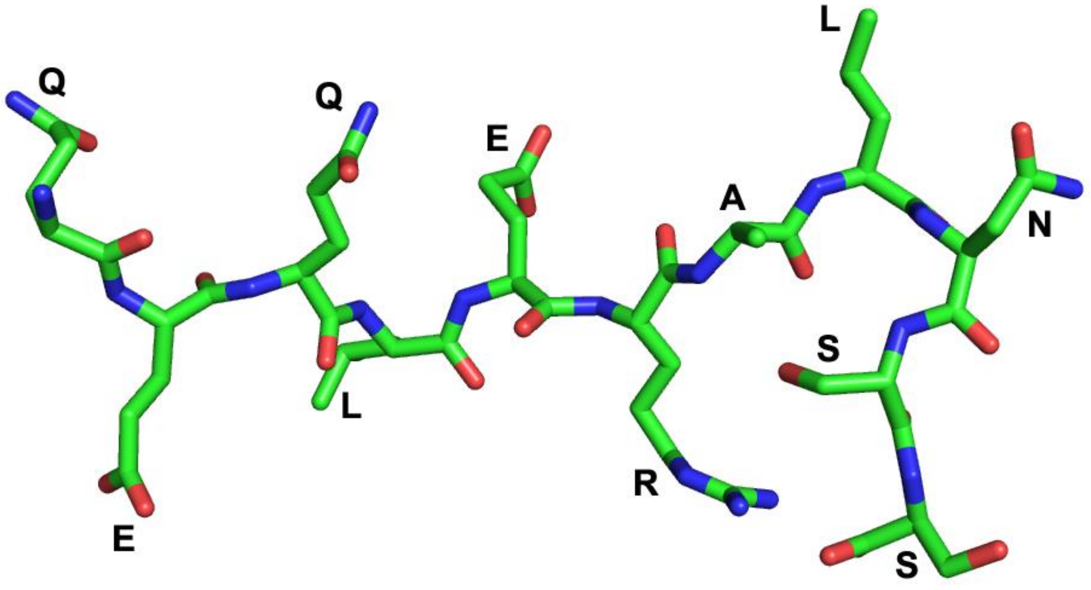

Supplementary Figure 1. Stick model of pHBSP. The amino acid sequence of pHBSP is: QEQLERALNSS. The initial peptide model was built using the PyMOL(TM) Molecular Graphics System (V1.7.0.0), then, the generated model was solvated in a periodic cubic box and filled with the TIP3 water. 1 ns of molecule dynamic simulation were performed after 2000 steps of molecule minimization in NAMD2.9. Carton, nitrogen and oxygen atoms are colored in green, blue and red respectively.

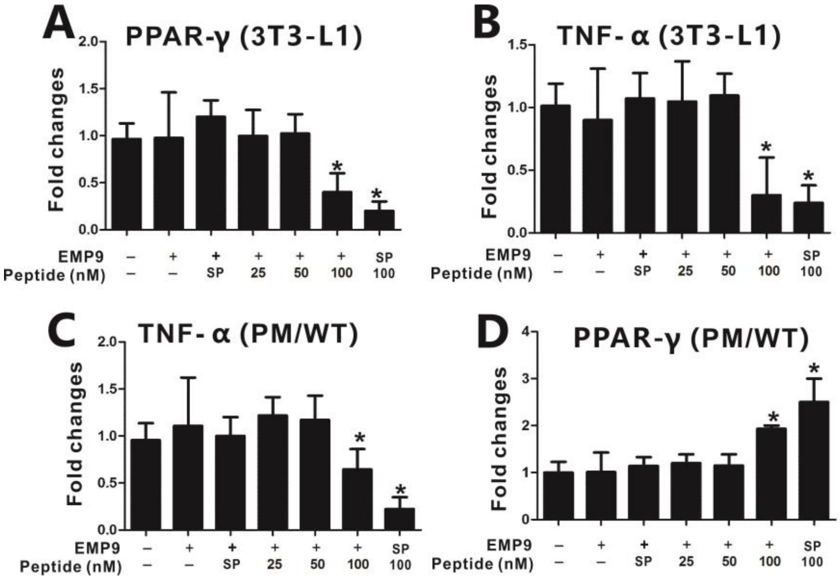

Supplementary Figure 2. 0.05mg/mL EMP9 couldn't completely deprive the anti-inflammatory function of pHBSP. A-B: Undifferentiated cells were induced to differentiate into mature 3T3-L1 pre-adipocytes by treatment with MDI for 8 hours followed by insulin supplementation, and data shows that administration of 0.05mg/mL EMP9 deprived the effect of 25 and 50nM pHBSP on PPAR-γ and TNF-α expression, while didn't deprive 100nM pHBSP on PPAR-γ and TNF-α expression in 3T3-L1 cells. **C-D:** Peritoneal macrophages were treated with 1μg/mL LPS followed by 0.05mg/mL EMP9 as well as various doses of pHBSP or PBS/scrambled peptide control intervention with for 4 hours, and data shows that administration of 0.05mg/mL EMP9 deprived the effect of 25 and 50nM pHBSP on PPAR-γ and TNF-α expression, while didn't deprive 100nM pHBSP on PPAR-γ and TNF-α expression in peritoneal macrophages. N= 3 per group; PM indicates peritoneal macrophages; SP indicates scrambled peptide; data are mean ± SEM; error bars indicate s.e.m. and significance is indicated by *P < 0.05 determined by one-way analysis of variance, followed by Dunnett's multiple comparison test.
